# THE EFFECT OF COGNITIVE TESTING ON STATE ANXIETY AND CORTISOL REACTIVITY IN YOUNGER AND OLDER ADULTS

**DOI:** 10.1093/geroni/igz038.2601

**Published:** 2019-11-08

**Authors:** MacKenzie L Hughes, Ann Pearman, Shevaun D Neupert

**Affiliations:** 1 Georgia Institute of Technology, Atlanta, Georgia, United States; 2 North Carolina State University, Raleigh, North Carolina, United States

## Abstract

Although it is well established that stress is negatively associated with cognitive functioning, less is known about age differences in the effects of stressors and anxiety on state anxiety and physiological reactivity (i.e., changes in cortisol). The current study examined state anxiety and cortisol reactivity during a series of cognitive tasks in a sample of younger (n=26) and older (n=29) adults. Participants completed the State-Trait Anxiety Inventory prior to cognitive testing and provided six salivary cortisol samples throughout one testing session: two cortisol samples prior to cognitive testing, three samples during testing, and one sample after testing. Six cognitive tasks were administered that measured attention span, declarative memory, and processing speed. Results indicated a significant interaction effect of age by time with younger adults’ cortisol linearly decreasing during the testing session and older adults’ cortisol showing a quadratic trend. A second interaction was found between age and state anxiety whereby older adults who reported more anxiety had higher cortisol levels during the cognitive testing session than both the older adults who reported low levels of anxiety and the younger adults. Only age (not cortisol or anxiety) was significantly related to cognitive performance. Results from this study suggest that standard cognitive testing could be anxiety producing for older adults, particularly for those who are already anxious. Future investigations should examine age-related differences in the processes linking anxiety and cortisol to specific types of performance, such as memory and attention.

